# Book Review

**Published:** 1931

**Authors:** 


### Reviews of Books

**Anatomy in the Living Model.** By D. Waterston, M.D., F.R.C.S.E., F.R.S.E. Pp. xvii., 255. Illustrated. London: Hodder & Stoughton. 1931. Price 25s.—The preface of this work affords no clue as to what particular section of the medical public it is addressed to. If it is to the average student of medicine, we can imagine his holding up his hands in horror at the very idea that, somewhere in his overcrowded curriculum, he must find time to study it, composed as it is of a mixture of various kinds of anatomy (systematic, surface, and what may be called artistic), with some physiology and comparative anatomy thrown in. What would a student say in private if, sometime presumably in his pre-clinical days, he were required to devote special attention to the study of the colour of the skin and the physiology—or is it pathology—of "goose-skin"? The Basle terminology is adopted, so we think it a pity to resuscitate the sub-costal plane as a means of sub-dividing the surface of the abdomen instead of the more desirable transpyloric plane of Addison; moreover, we must surely make up our minds once for all what plane we are going to use in dividing the abdomen vertically. Are not the pure anatomists agreed that the lateral vertical plane is to be preferred to the mid-Poupart or the mid-clavicular? Perhaps these are not points of great practical importance, but the student must know just where he stands, with examiners at any rate. The clinician will disagree with the statement that the umbilicus is of little value as a landmark: he uses it every day, and its position is quite sufficiently fixed for his purpose. Seeing that the book is so largely devoted to the study of surface structures, it is curious that the breast seems to have escaped notice. Altogether rather a disappointing book, whose appeal we fear will be to a somewhat limited circle of readers.

**The Treatment of Asthma.** By A. H. Douthwaite, M.D., F.R.C.P. Pp. viii., 164. London: H. K. Lewis & Co. Ltd. 1930. Price 7s. 6d.—In his preface the author states that the object of the book is to present an epitome of modern medical knowledge on asthma with special reference to treatment. He has certainly succeeded. The reader has a feeling that the subject is presented dispassionately without overstressing particular aspects of the condition. The division of asthma into primary and secondary is of great practical importance, and affords a sound method of approach for both diagnosis and treatment. The author's description of physical signs, in which he clearly differentiates early and late cases, the latter associated with definite organic changes in the chest, is most useful and, later in the book, affords a basis of treatment by physiotherapy, a method which is considerably neglected. The book contains a useful summary of the recent work on metabolism, allergic reactions, and bacteriology. Prominence is given to the recent work of Oriel, which seems likely to prove of considerable diagnostic value. The therapeutic section of the book contains a well-balanced account of the accepted methods of treatment. Prominence is given to the difficulties of desensitization, and tribute is paid to the importance of psychotherapy; but the author lays special stress on the great importance of remedial exercises and physiotherapy from the prophylactic point of view, in early cases, in order to prevent the development of grave organic changes in the thorax which must develop sooner or later in neglected cases.

**Therapeutic Uses of Infra-Red Rays.** By W. A. TROUP, M.C., M.B. Pp. xii., 57. Illustrated. London: Actinic Press Ltd. 1930. Price 5s. 6d.—So much has been written about the uses of ultra-violet rays in the treatment of diseased conditions, that it has been for several years the common practice to utilize this form of energy in some forms of tuberculosis and other conditions as a matter of course. But little has been heard of the use of infra-red rays. Dr. Troup has, therefore, produced a useful little monograph which serves to introduce to us the various uses to which these rays may be put. Heat in some form or another has been recognized as a beneficial agent, and various methods of utilizing heat, from poultices to diathermy, have been in vogue. Now it has been found that a local, penetrating heat ray can be at the command of the practitioner from sources actuated by electricity and easily manipulated. Dr. Troup describes the various lamps which are in the market and their field of usefulness. He tells us of the action of these rays in arresting and relieving pain, of the absorption of effused products round sprains and injuries, and the reconditioning of paretic muscles. If all that he claims for this agent is true, then none of us can afford to do without one of these infra-red lamps, and Dr. Troup's book to guide us in its use. The book is very short and concise and takes but an hour to read, but it is pregnant with suggestions of a helpful kind, and we welcome it as the first of its kind in England.

**Emergency Surgery. Vol. I: Abdomen and Pelvis.** By HAMILTON BAILEY, F.R.C.S. Pp. xviii., 380. 324 illustrations. Bristol: John Wright & Sons Ltd. 1930. Price 25s.—The present volume, which deals with the emergency operations on the abdomen and genito-urinary system, has compressed within its handy size more wisdom than is found in most of the far larger text-books on the subject. Moreover, there is a most admirable clarity of diction and illustration. The author has conferred a great boon on the profession by a discriminate collection of the really useful points from the countless monographs and latest practice of our day. These he has related to his own experience, and the result is a convincing compendium. Where the matter and the manner of its presentation are so much in advance of current works, it is hard to select chapters either for special praise or for criticism. The author's emphasis appears placed somewhat too much on the expectant treatment of appendicitis, so that the inexperienced might neglect to operate as soon as is desirable. This and a few minor points of technique are negligible features compared with the general merits of the work: it makes clear the indications for emergency surgery, and still clearer the steps of each operation; no book gives such lucid and graphic descriptions of operative method, and so many hints to overcome the little hitches that beset the path of the young surgeon. The illustrations are extremely well chosen, and are reproduced with the excellence that we have learnt to expect from the firm of Wright and Sons. There is an adequate index.

**Stepping Stones to Surgery.** By L. BATHE RAWLING, F.R.C.S. Pp. xv., 227. Illustrated. London: H. K. Lewis & Co. Ltd. 1930. Price 12s. 6d.—This book is designed, in the words of the author, to provide a bridge for the gap which separates anatomy and surgery. It is a combination of applied anatomy and elementary surgery and is arranged in thirty-one chapters, each one of which deals with a separate region of the body, and commences with a short description of a clinical case of some disease or injury of the part under consideration. This plan makes for readability, a great desideratum in a students' text-book on a subject which is apt to be shirked, although it tends to make the work somewhat scrappy and disjointed. It is doubtful if every surgeon will accept the statement in the last sentence of the book, that the subscapular glands are rarely involved in carcinoma of the breast. A slang word like "innards," even in inverted commas, seems rather out of place in a scientific work. There are a number of misprints. These are small matters in a book which should be of great use in acquiring knowledge of a difficult but important subject. The index and the illustrations are adequate.

**The Student's Handbook of Surgical Operations.** By Sir FREDERICK TREVES, Bart., G.C.V.O., C.B., LL.D., F.R.C.S., and CECIL P. G. WAKELEY, F.R.C.S., F.R.S.E. Fifth Edition. Pp. xii., 535. Illustrated. London: Cassell & Co. Ltd. 1930. Price 10s. 6d.—This book has been deservedly popular ever since its first appearance in 1892. By skilful pruning and grafting Mr. Wakeley has contrived to bring it up to date, while preserving its convenient size and arrangement. Some illustrations have been omitted and others added, but it is remarkable how many of the originals still fulfil their purpose and consequently remain. Still more obsolete or obsolescent procedures might have been omitted, and further modern technical improvements might well have been incorporated. Amongst the last are the use of intratracheal anæsthesia and of the "blood-sucker" in mouth operations; the diathermy knife; and fascial sutures in the repair of large hernias. One paragraph three-quarter way down page 395 is unintelligible and needs re-writing, but there are very few typographical errors. We would point out that hemithyroidectomy is seldom a sufficiently drastic operation for the cure of exophthalmic goitre. These are, however, minor defects, and there are many useful additions, notably a clear and simple chapter on the uses of radium in the treatment of cancer. The present edition is likely to prove as helpful to and as popular with the student of to-day as former editions have in the case of his predecessors.

**Congenital Club Foot.** By E. P. BROCKMAN, M.Ch., F.R.C.S. Pp. viii., 110. Illustrated. Bristol: J. Wright & Sons Ltd. 1930. Price 10s. 6d.—This monograph was awarded the "Robert Jones" Gold Medal for 1928, and it is the only up-to-date book to tackle the problem of congenital talipes equino-varus. The opening chapters contain an interesting review of the literature with an accurate description of the nature of the deformity. Chapter IV. deals in a masterly manner with the pathological anatomy of the common variety of club foot. The author points out that there is essentially a failure of development of all the structures of the foot, with a congenital subluxation of the astragalo-calcaneo-scaphoid joint. Failure to correct this subluxation is the prime cause of the so-called relapse cases. The author is of the opinion that the deformity should be dealt with by open operation in all cases not readily responding to manipulative measures and he describes a new operation for this. His operation entails an extensive dissection of the structures of the sole of the foot, whereby the contracted muscles are lengthened and the scaphoid separated from the sustentaculum, thus enlarging the socket for the head of the astragalus. From the description, the operation appears unnecessarily severe; but, as the author points out, the trauma is probably not greater than that produced by repeated violent manipulations. We have no experience in his own operation, but the case reports are its justification. The illustrations have been well chosen and excellently reproduced. The book is readable and well balanced, and records yet another advance in orthopædic surgery.

**The Treatment of Chronic Deafness by the Electrophonoide Method of Zund-Burgüet.** By G. C. CATHCART, M.D. Second Edition. Pp. xiii., 111. London: Oxford University Press. 1931. Price 5s. **Deafness and its Alleviation by Operation.** By V. NESFIELD, F.R.C.S. Second Edition. Pp. vii., 169. Illustrated. London: H. K. Lewis & Co., Ltd. 1931. Price 10s. 6d.—The first of these two books is a reprint of the edition recently noticed in these columns. The author takes us to task for seeing a resemblance between his cherished but mysterious machine and Abram's box, so we must tread warily. The essence of our criticism was that the method involves from 12 to 72 or more "treatments" with an intricate and expensive apparatus, and that the claim of absolute and unvarying accuracy for the only "test"—the author's voice—must prove a σκάνδαλον. This edition shows no improvement on the former: even the quotation from Fitzgerald's *Omar Khayyám* retains its Limerick metre. The second work seems at first sight to be written in the advocacy of Phenolaine, eight of the eleven local applications advised for nose and ear and numerous other passages recommending this proprietary preparation. And we cannot but recall the laudatory mention of the same drug in *Nesfield's Ophthalmic Surgery*. For our author is no narrow specialist ; his activities have included those of Prospector (*sic*) to the R.C.S., bacteriologist, public analyst and "inventor of the chlorine and iodine processes of sterilizing water." A cursory account of the anatomy and physiology of the ear leads us via some very speculative pathology to a far from clear description of "The Operation," apparently that usually associated with the name of Mr. Chas. Heath. A series of case records completes the volume. Of the 20 illustrations which serve to break up the text five are catalogue blocks of common instruments : the remainder indifferent line drawings, eight being copies of text-book plates so well known that the author only deems it necessary to "acknowledge" two. Repetitions, even of whole pages, spelling mistakes and grammatical errors (*e.g.*, foramen ovalis) are numerous. This also is a second edition : how wonderful are the ways of Allah !

**Pharmacopœia of the Central London Ophthalmic Hospital.** Pp. 30. London : H. K. Lewis & Co. Ltd. 1930. Price 2s.—This small volume gives a full list of the preparations used in the practice of an ophthalmic hospital. It should be a handy volume for those who wish to refer to the doses of drugs which are not frequently used in every-day practice, and of the strengths of the drugs and lotions which are of value in dealing with diseases of the eye.

**Royal Army Medical Corps Aids to Dispensing.** By Staff-Sergt. G. ASHTON, R.A.M.C. Pp. xii., 232. Aldershot : Gale & Polden Ltd. 1930. Price 6s. 6d.—Staff-Sergeant Ashton has been modest in the title he has given to this book, for it is a comprehensive if condensed summary of materia medica, the B.P., pharmacy, dispensing, and poisons and antidotes. Regarded as the text-book for Army compounders, it indicates a very great advance indeed on the pre-war standard of knowledge expected from them, and is put together in an excellent form for the student. The materia medica under each drug gives the macroscopic characters, doses, therapeutic action and B.P. preparations with their doses and strengths. A series of dose tables classifying the chief doses in order of magnitude precedes a very efficient synopsis of B.P. preparations with excellent tabular arrangement. A section on weights and measures introduces the reader to practical dispensing. One is inclined to regret the tables on pp. 130–131, in which the author gives methods for the elaborate and unnecessary conversion of metric formulæ into apothecaries' formulæ, when it is so desirable to familiarize the student with the metric system as one for every-day use. Better to teach the student to make 200 mils. of Tr. Opii Ammon. than to make long calculations of conversion in order to make ℥vi of the preparation. The section on practical pharmacy is very good, and deals with most branches of the art. The sodium chloride might well be omitted from the paraffin spray on p. 165, and alkaloid cocaine should replace the hydrochloride. A useful chapter on poisons concludes a very practical book, an Army compounder's *multum in parvo*.

**Clio Medica: a Series of Primers on the History of Medicine.** Editor: E. B. KRUMBHAAR, M.D. New York. Paul B. Hoeber. 1930. Price $1.50 each volume. 1, **The Beginnings: Egypt and Assyria,** by W. R. DAWSON, F.R.S.E.; 2, **Medicine in the British Isles,** by Sir D'ARCY POWER, K.B.E., F.R.C.S.; 3, **Anatomy,** by GEORGE W. CORNER, M.D.; 4, **Internal Medicine,** by Sir HUMPHRY ROLLESTON, Bart., K.C.B., M.D.; 5, **Physiology,** by JOHN FULTON, Ph.D., M.D.—We have received the first five volumes of a series of handbooks which, under the general title of *Clio Medica*, aims at presenting in a concise and readable form a number of special phases in the history of medical science. The publishers, realizing that small, easily portable booklets have, since the times of the Aldine and Elzevir editions, been popular with busy students and intelligent readers, offer in this series books for the coat pocket or bedside reading. But a wonderful kernel of information is provided in each of the booklets. The names of the authors is sufficient guarantee that the subjects will be adequately and accurately dealt with. Dawson takes the beginnings of medicine as they developed in Egypt and Assyria from a welter of magic, superstition and priestcraft into systematic and rational therapeutics. Primitive and simple as their therapeutics were, we are reminded that anatomy arose out of the embalmer's art, whilst a number of the most valuable drugs in modern pharmacy were first put to medicinal uses by the Egyptians. The Assyrians possessed a recognized medical profession whose members employed drugs in their prescriptions which are, in the main, wholesome and rational, even though incantations and charms formed an integral part of the ritual of treatment. Sir D'Arcy Power calls his account of Medicine in the British Isles "the barest outline," but he contrives to give a delightful thumbnail sketch of the history of the medical corporations which have governed medical education and practice in this country. Corner, on the history of Anatomy, guides the reader rapidly through the earliest phases in Greece and Rome to the Middle Ages, and then gives an admirable description of the rise and expansion of modern anatomy from Vesalius to histology, embryology and neurology. He even finds space to complain that teachers still try to compel each generation of students to memorize as nearly as possible everything gained since Vesalius and advocates the use of the great text-books for reference rather than memorization, so that study may be directed to principles and methods instead of mass assembly of facts. His illustrations are numerous and well-selected. Sir Humphry Rolleston deals with Internal Medicine in a more or less biographical manner, claiming, with justice, that the study of the published works and the individuality of the prominent figures of successive periods furnishes material for a good general survey of progress. Fulton takes a somewhat similar view of Physiology, weaving into his story significant details of the lives of those men who have contributed most to the advancement of our knowledge. When he comes to modern trends he expresses some preference for physiologists who will make the objective of their science the human body, and endeavour to comprehend *its* functions rather than those of frogs and cats and apes. "It were better," he says, "that many of the so-called 'general' physiologists recognize that they are not physiologists at all," and offers to them as more correct descriptions the titles of "biophysicists," "protein-chemists" or "electro-chemists." Thus it will be appreciated from this short review of five delightful volumes that the authors each in their own way have made the dry bones of history live, reminding us again and again of the goodly heritage of medicine to which we are heirs.

